# Cost Effectiveness of TNF-*α* Inhibitors in Rheumatoid Arthritis

**DOI:** 10.1155/2013/581409

**Published:** 2013-11-13

**Authors:** Cynthia Said, Bernard Coleiro, Maurice Zarb Adami, Lilian M. Azzopardi, Anthony Serracino Inglott

**Affiliations:** ^1^Department of Pharmacy, Faculty of Medicine and Surgery, University of Malta, Msida MSD 2080, Malta; ^2^Rheumatology, Department of Medicine, Mater Dei Hospital, Msida MSD 2090, Malta

## Abstract

*Background.* TNF-*α* inhibitors have shown to be effective in reducing disease activity and improving the quality of life. Due to the high costs associated with acquisition of this treatment, this study was undertaken to evaluate the ICER of TNF-*α* antagonists (etanercept, adalimumab, and infliximab) in improving the quality of life. *Methods.* The HAQ and SF-36 were administered at phases 1, 2, and 3, in order to assess the improvement in the QOL. Suppression of disease activity was assessed through the DAS-28. *Results.* Statistically significant improvements (*P* < 0.05) were noted for the SF-36 and HAQ after 3 months and for the DAS-28 after 6 months of TNF-*α* inhibitor therapy. The mean ICER per 10% improvement in the HAQ, DAS-28, and SF-6D were €1976.5, €2086.5, and €2316.4, respectively, following 6 months of TNF-*α* intervention. Most favorable ICERs were reported from a patient who had to undergo surgical intervention whilst on DMARD therapy. *Conclusion.* Significant improvement was observed in patients' quality of life, after a short timeframe of 6 months. Such data is useful information in the light of convincing policy makers, in terms of providing access to the medications to individual patients on national health service schemes.

## 1. Introduction

Rheumatoid arthritis (RA) is a progressive, inflammatory disease which is characterised by inflammation of the joint synovium that could ultimately progress to joint destruction [1, 2]. Due to its chronic, immune-mediated course, long-term treatment with immune-modulatory drugs is generally required [3]. This disabling condition, is thought to affect 0.3–1.2% of the worldwide population [4]. Uncontrolled RA results in progressive joint destruction and functional decline [5]. This disabling condition imposes substantial economic burden through the decreased quality of life (QOL) and loss of productivity [6]. 

Recent advances in biotechnology and pathogenesis of RA have led to the discovery of biological DMARDs [6]. Biological agents inhibit pro-inflammatory cytokines which are believed to have a crucial role in the inflammatory process within the synovial joint [7]. TNF-*α* inhibitors have proved their clinical efficacy and raised the previous goals of RA treatment [5, 8]. Clinicians nowadays aim to achieve low disease activity or preferably remission and not merely slowing the progression of the disease and controlling symptoms [9]. The discovery of biological agents has led to a drastic shift in the therapeutic approach to RA, leading to a better QOL [10]. 

Yet, these breakthrough drugs are associated with high procurement costs. This ultimately increases the financial burden RA imposes on society [4, 5]. Such a scenario has elicited the need to carry out pharmacoeconomical assessments in order to inform policy and decision makers of the cost-effectiveness of the biological DMARDs [5, 8]. Hence, this study was undertaken to determine the improved QOL and the incremental cost-effectiveness ratio (ICER) involved in treating Maltese patients suffering from resistant RA, with TNF-*α* inhibitors.

## 2. Materials and Methods

Data collection used to conduct this 6-month study was carried out at the Rheumatology Outpatient Clinic at Mater Dei Hospital. Patients were eligible to participate in the study provided that they had been diagnosed with RA according to the 1987 ACR classification criteria, failed to achieve a low disease activity despite DMARD/s therapy; and were switched onto a TNF-*α* inhibitor (etanercept, adalimumab or infliximab). Patients were not eligible to participate if pregnant or planning to conceive suffering from TB or hep B; they are diagnosed with juvenile chronic arthritis, ankylosing spondylitis, osteoarthritis, psoriatic arthritis, and/or any other rheumatological condition.

Following approval from the Maltese Research Ethics Committee Board, patients identified for participation were briefed on the purpose of the study. A signed consent form was obtained from every patient who accepted to participate, out of their own voluntarily will. 

The disease-specific Health Assessment Questionnaire (HAQ) was chosen as an outcome measure tool to assist in the evaluation of the functional improvement experienced by patients that were switched onto TNF-*α* inhibitor therapy. The HAQ is a reliable tool used by various studies to assess daily activities, namely, dressing, grooming, eating, walking, hygiene, and so forth. Each of these items is given a score from 0 (without any difficulty) to 3 (unable to perform) reflecting the patient's ability in performing a particular activity [11].

The generic SF-36 is a health status questionnaire which has become a predominant tool in assessing various medical interventions consisting of 8 domains and 2 summary scores [12]. Unlike the HAQ, the higher the score obtained in each of the SF-36 domains, the more it indicates a better health status in the respective domain. 

The DAS-28 was used as a clinical outcome measure tool in order to monitor disease activity with biological intervention. The DAS-28 generates a continuous scale (0 to 9.4) through the assessment of tender joints (0–28), swollen joints (0–28), erythrocyte sedimentation rate (mm/hr), and the Visual Analogue Scale (0–100) [13]. 

This prospective study, carried out between 2010 and 2011, had a time phase of 6 months, during which patients were assessed 3 times through the SF-36 and HAQ. At phase 1 (t—0 months), patients were still being treated with conventional DMARD therapy. Failure to achieve the desired outcome and following discussions with the rheumatology consultant, patients were identified as suitable candidates for initiation of TNF-*α* inhibitor therapy. Subjects who accepted to participate in the study were interviewed by the investigator using the HAQ and SF-36. Raw data obtained for both the SF-36 and the HAQ were inputted in a Microsoft Excel Database. The final HAQ score and summary scores for every SF-36 domain were calculated. At phase 2 (t—3 months after initiation of TNF-*α* inhibitor therapy), participants were reinterviewed using the same questionnaires. At phase 3 (t—6 months), data from the SF-36 and HAQ were again collected following 6 months of TNF-*α* inhibitor therapy. 

The SF-36 scores were converted into SF-6D scores using the algorithms provided by the University of Sheffield. The Excel program based on nonparametric Bayesian preference weights was chosen since the non-parametric model has shown added benefits in the predictive capacity of the model over the parametric random effects model [14]. The raw scores for every item within the SF-36 for all the 13 patients were inputted in the Excel programme which generated utility values, anchored at 0 for dead and 1 indicative of full health [15].

During both phases 1 and 3, patients were examined by the rheumatology consultant and assigned a DAS-28 score as a measure of disease activity. In both phases, medical case notes of patients were reviewed and related data were collected, namely, DAS-28 scores, treatment regimens, adverse events, history of hospitalisation during the study period, and surgeries performed related to RA. 

Data collected from the HAQ and SF-36 questionnaires were analyzed statistically using SPSS version 18.0. The one-way ANOVA test was used to compare the mean scores obtained during phase 1, 2, and 3 for both the HAQ and the SF-36. Scores obtained in the HAQ and SF-36 were also analysed using the post hoc Tukey's test where pairwise comparison between the mean rating scores of any two visits was carried out. 

The costs of TNF-*α* inhibitors, DMARDs, and glucocorticoids involved in the study were obtained from the Maltese Government Health Pharmaceutical Services (GHPS). Treatment regimens for every patient were obtained by scrutiny of patients' notes prior to initiation of TNF-*α* inhibitor therapy (Treatment B) and after 6 months of biological therapy (Treatment A). Costs of Treatment B included the costs of DMARDs and concomitant steroids for a period of 6 months prior to phase 1. Hospitalization and hip replacement surgery costs were included, assuming that the use of a TNF-inhibitor would have decreased the need of hospitalization and hip replacement surgery. Currently, Maltese patients, who have an inadequate response to multiple DMARDs and suffer from moderate to severe RA, are considered for biological agent therapy as recommended by the ACR guidelines [8]. Prior to commencement of a TNF-*α* inhibitor, patients participating in the study had been on the same nonbiological DMARD/s for at least 6 months. The direct costs of Treatment A included the cost of the biological agent being administered, any concomitant DMARD, and steroids for a period of 6 months.

The economic evaluation used in this study was a cost effectiveness analysis (CEA) which combines the difference between the costs of treatments with improvement achieved through biological intervention. The difference in costs between treatments was established by subtracting the cost of Treatment B from Treatment A for every patient. The improvement in the HAQ was chosen as a unit of health benefit since the HAQ is a strong predictor of the functional disability observed in patients with RA through loss of productivity [5, 16]. The ICER per unit of improvement in HAQ was calculated using the following formula:

(1) ICER  per  unit  improvement  in  HAQ =(Cost  of  Treatment  A−Cost  of  Treatment  B)  ×(HAQ  score  on⁡  Treatment  B    − HAQ  score  on⁡  Treatment  A)−1.

The DAS-28 score was also used as a unit of health benefit, as it highly reflects the disease activity and is thus useful in indicating the suppression of disease activity following a therapeutic intervention [13]. The ICER per unit of improvement in the DAS-28 through biological intervention was calculated using the following formula:

(2) ICER  per  unit  improvement  in  DAS-28 =(Cost  of  Treatment  A−Cost  of  Treatment  B)  ×(DAS-28  score  on⁡  Treatment  B    − DAS-28  score  on⁡  Treatment  A)−1.

Both the HAQ and the DAS-28 give an ordinal scoring system which is often criticized if used as a unit of health benefit to express the incremental ICER [4]. The SF-6D provides scores on a cardinal scale from 0 to 1 with equal grades and can therefore be used in assessing cost effectiveness of a health intervention [17]. 

Hence, the ICER was expressed in terms of unit improvement in the SF-6D using the following formula:

(3) ICER  per  unit  improvement  in  SF-6D =(Cost  of  Treatment  A−Cost  of  Treatment  B)  ×(SF-6D  utility  index  on⁡  Treatment  B    − SF-6D  utility  index  Treatment  A)−1.

Comparison between resulting ICERs using the 3 different methodologies could not be made since the (i) HAQ gave scores ranging from 0 to 3, (ii) the DAS-28 scores ranged from 0 to 9.4, and (iii) the SF-6D ranged from 0 to 1. This has prompted the researcher to devise a scoring scheme ranging from 0 to 10 in order to compare ICERs obtained over a 6-month period. The resulting ICERs from all 3 different methodologies were calculated and expressed in terms of this scoring scheme. One-way Anova testing was conducted on the resulting ICERs in order to assess significance between outcome health benefits chosen.

## 3. Results

13 patients were recruited within the study out of which 85% were females, with a mean age of 54 years. Patients had been suffering from RA, prior to initiation of TNF-*α* inhibitor therapy for a mean of 11 years ranging from 4 to 31 years. 

**Figure 1:**
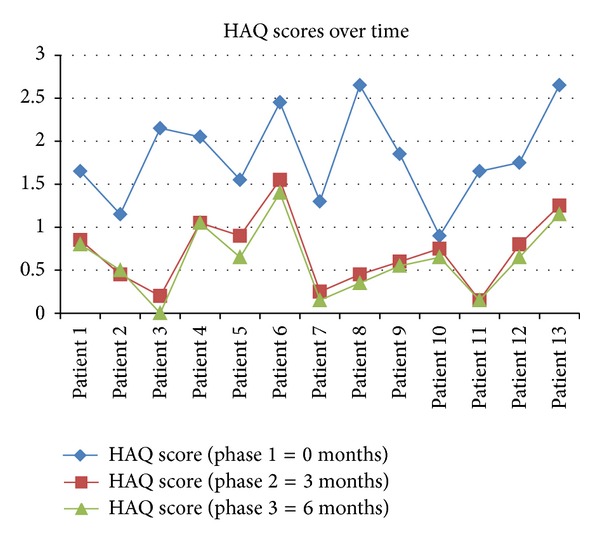
Illustration of the HAQ scores reported by patients at baseline, at 3 months, and 6 months following TNF-*α* inhibitor therapy.

As shown in Figure 1, the mean HAQ scores achieved in phase 1 were the highest indicating the functional disability experienced despite conventional DMARD therapy. Substantial improvement was noted after 3 months of biological therapy. Progress achieved was sustained and continued to improve during the last 3 months of the study, yet to a lesser extent than in the previous 3 months. 

Table 1 shows that all activities assessed within the HAQ have improved significantly (*P* < 0.05) between phase 1 and 2. The mean difference in the HAQ scores obtained between phase 2 and phase 3 was not statistically significant (*P* > 0.05). During the whole study, the mean scores obtained were statistically significant (*P* < 0.05). This result indicates that significant improvement is observed as early as 3 months after initiation of TNF-*α* inhibitor therapy.

Table 1 shows the difference in mean scores obtained between phases for every activity assessed within the HAQ and their relative level of significance (significance noted at *P* < 0.05*).

**Figure 3:**
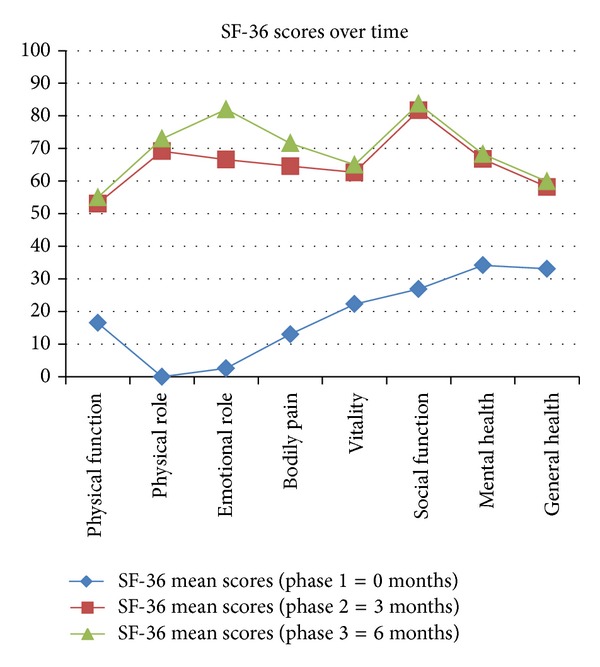
Illustration of the scores for every domain within the SF-36 at baseline, at 3 months, and 6 months following TNF-*α* inhibitor therapy.

As noted from Figure 3, patients reported low functional status in the SF-36 whilst on DMARD therapy. During phase 2 and 3, all patients reported an improvement in the functionality for all domains within the SF-36. The greatest improvement was observed in the physical and emotional role between phase 1 and 2. The least improvement reported was for general health. 

Every SF-36 domain improved significantly between phase 1 (baseline) and phase 2 since *P* values obtained were <0.05 level of significance (Table 2). As observed with the HAQ scores, *P* values between phase 2 and 3 for every SF-36 domain were not significant (*P* > 0.05). SF-36 domains improved significantly after 6 months of biological intervention between phase 1 and 3 (*P* < 0.05). Lower *P* values were obtained for the ER and BP when compared with other domains between phase 2 and 3, thereby indicating continuous improvement during the latter part of the study.

Table 2 shows the difference in mean scores obtained between phases for every SF-36 domain and their relative level of significance (significance noted at *P* < 0.05*).

**Figure 2:**
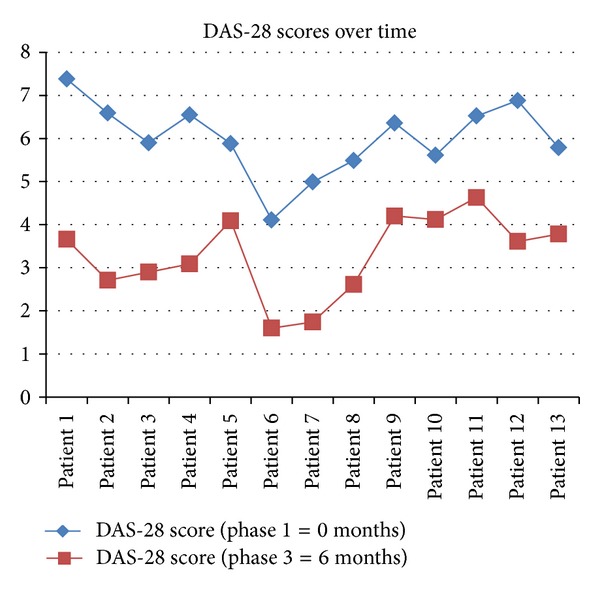
Illustration of the DAS-28 scores reported by every patient at baseline (prior to initiation of TNF-*α* inhibitor therapy) and at phase 3 (6 months after initiation of TNF-*α* inhibitor therapy).

During phase 1, as observed in Figure 2, 85% of patients (*n* = 11) scored higher than 5.1 indicating a high disease activity according to the EULAR criteria. In accordance with the EULAR criteria response, a decrease of 1.2 from the initial DAS-28 score has been found to indicate a significant change and is thus considered as a good response [13]. After 6 months of biological intervention, the DAS-28 decreased significantly (>1.2) for all 13 patients. 31% of patients (*n* = 4) scored <3.2 in the DAS-28 which corresponds to low disease activity whilst 15.4% (*n* = 2) scored <2.6 which is compatible with disease remission. 54% of patients (*n* = 7) scored values compatible with moderate disease activity (>3.2 ≤5.1) [13].

A slight improvement in the HAQ score despite biological intervention results in a high ICER (Table 3). Patient 10 experienced a minimal improvement in the quality of life which gave an ICER of €26,030 over a period of 6 months. The rest of subjects (*n* = 12) experienced a much lower ICER due to a higher improvement recorded in the quality of life. Patient 13 had undergone a hip replacement surgery prior to phase 1 and hence gave a more favorable ICER, due to a lower discrepancy between the costs of treatments. In order to improve by 10% in the quality of life as measured by the HAQ, an average addition of **€1,977** is incurred over a period of 6 months.

Table 3 shows the ICERs per unit improvement and per 10% improvement in the HAQ following 6 months of TNF-*α* inhibitor therapy.

The least reduction in disease activity was again reported by patient 10 (Table 4). This eventually resulted in the highest ICER per unit of improvement in the DAS-28. The lowest resulting ICER was incurred again by patient 13. For every unit of improvement in the DAS-28 (reduction in disease activity by 1 on a scale from 0 to 9.4) an increase of €2,200 was incurred over a period of 6 months. The average reduction in disease activity (DAS-28) was of 2.71, which is considered as a significant good response (>1.2) [13]. In order to achieve a reduction of 10% in the DAS-28 score (i.e., 0.94), it would cost the Maltese Government an additional **€2,087**.

Table 4 shows the ICERs per unit improvement and per 10% improvement in the DAS-28 following 6 months of TNF-*α* inhibitor therapy.

An average patient, following 6 months of TNF-*α* inhibitor, gained a 0.27 improvement in the utility health index, the SF-6D. Theoretically, an ICER of € 23,164 is incurred for a patient to regain a perfect quality of life (1) from a state (0) equivalent to death (Table 5). An improvement of 0.1 as assessed through the SF-6D would cost the payer an additional **€2,316**. 

Table 5 shows the ICERs per unit improvement and per 10% improvement in the SF-6D following 6 months of TNF-*α* inhibitor therapy.

No significant difference (*P* > 0.05) was noted between the 3 different methodologies used to calculate the ICER (Table 6). The choice of the SF-6D yielded the higher ICER when compared to resulting ICERs in terms of the HAQ and DAS-28.

Table 6 shows the level of significance (significance noted at *P* < 0.05) and standard deviations between the HAQ, DAS-28, and the SF-6D.

## 4. Discussion

Results obtained within this study highlight the evidence of the significant improvement gained in the QOL caused by this disabling condition in the initial treatment phases. Various studies have used the HAQ to shed light on the quality of life and physical disability endured by patients [18]. At baseline, patients reported a score ranging between 1.3 and 2.65. In a study by del Moral et al. [16], an HAQ score >1.5 was correlated with a significant number of working days absenteeism (>50%) than those scoring <0.5. This implies that costs are further increased by the loss of productivity experienced by patients. During this study, indirect costs such as loss of work were not included since a high percentage of the subjects were housewives and thus it would be very difficult to quantify the work carried out or the lack of it. Following 3 months of treatment with TNF-*α* inhibitors, scores ranged from 0.2 to 1.5, indicating a considerable significant improvement (*P* < 0.05). Scores continued to decrease furthermore during the last 3 months of the study yet significance was not noted (*P* > 0.05).

Apart from the functional impairment assessed within the HAQ, which hinders patients from their daily activities, RA affects considerably the vitality, emotional wellbeing, and mental health [19]. This has been confirmed by the low mean scores reported in the study for all domains within the SF-36 at phase 1. Despite multiple DMARD therapy and concomitant glucocorticoids, patients experienced the worst scores for the physical role and emotional role. All domains improved significantly after 3 months of TNF-*α* inhibitors (*P* < 0.05). In the latter half of the study, significance was not noted since scores achieved were similar to phase 2 apart from the emotional role which continued to improve (*P* > 0.05). This is in concordance with the findings from the HAQ which suggests that a “plateau” is reached after 3 months of biological intervention. Such an observation highlights the need for long-term studies, in order to assess whether improvement gained is sustained and to what extent. Findings from this study are in accordance with results obtained from the generic SF-36 questionnaire in a study conducted on a Spanish population [20]. Findings from both HRQOL questionnaires used indicate the clinical effectiveness of TNF-*α* antagonists in achieving a better QOL, as documented in various studies [20, 21].

The DAS-28 score used a clinical marker to monitor the disease activity after intervention with TNF-*α* inhibitors. At phase 1, 85% of subjects were experiencing a high disease activity (>5.1) as devised by the EULAR criteria. Baseline DAS-28 results further support the resulting disability and compromised quality of life reported in the HRQOL questionnaires. Following 6 months of biological intervention, prevalence of DAS-28 <3.2 was 31% (*n* = 4) which is compatible with low disease activity. All patients (*n* = 13) experienced significant reduction in disease activity (>1.2) as defined by the ACR/EULAR criteria. ACR/EULAR remission (<2.6) was met by 15.4% (*n* = 2) of subjects. Abalos Medina [20] reported a higher prevalence of ACR/EULAR remission, yet this must be interpreted in the light of the limited number of participants and length of the current study.

Recent therapeutic strategies in RA aim to slow down disease progression and reduce functional impairment [22]. TNF-*α* antagonists are the forerunners in achieving such a therapeutic goal as they have been proven to suppress disease activity [1]. Since the procurement of TNF-*α* antagonists is associated with incredibly high costs when compared with conventional DMARD therapy, economic evaluations are essential as benefit from biological treatment is evident, but financial constraints limit its availability [23]. The HAQ was chosen to assess the improvement achieved through biological intervention, since a close correlation was reported between HAQ and health care costs [24–26]. A unit of improvement in the HAQ (any 1 unit difference between 0 and 3) was chosen as a health outcome benefit in order to assess the ICER. The resulting ICERs per unit of HAQ varied between €1,201 and €26,030 with a mean of €6,588. Discrepancy between costs of treatment was offset by hip replacement surgery for patient 13 which resulted in a more favourable ICER. Such result is enlightening as it demonstrates that TNF-*α* antagonist therapy is more likely to be cost effective in patients with severe functional disability, who may ultimately require surgical interventions. The least favourable ICER was achieved by patient 10 who experienced the least improvement in the HAQ (gain of 0.25). In such a scenario, it is evident that the disease is resistant despite biological treatment and alternative treatments should be initiated in order to gain a better control of the disease and consequently QOL. Kobelt et al. [27] described a similar situation where direct costs with a score >2.6 was approximately twice the direct costs for patients achieving a score less than 0.6. This highlights the need for effective treatment in order to achieve maximal improvement in QOL and hence diminish the costs incurred. 

The DAS-28 is useful in indicating the suppression of disease activity with biological intervention [13]. Such measure of health benefit was chosen as it is free of any bias from both the researcher and the patient. Subjects achieved an average significant reduction of 2.71 (>1.2) following 6 months of treatment. The highest ICER in terms of DAS-28 was again reported by patient 10 which resulted due to a minor improvement reported in the DAS-28. The resulting ICERs ranged from € 897 to €4,368 with the lowest ICER reported by patient 13. 

The ICER per unit of improvement in the SF-6D gave an average of €23,164 which is extremely higher than the ICERs per unit of improvement in the HAQ and DAS-28. Yet such a high cost resulted since it accounts for a transition between death and a perfect quality of life. All ICER results were expressed in terms of the same scoring scheme to be able to compare results. For every 10% improvement in the SF-6D, a cost of **€2,316** was incurred after 6 months of TNF-*α* antagonist intervention. Statistical significance was not noted (*P* > 0.05) between results obtained per 10% improvement in the respective health outcome. This could be either due to the limited number of observations (*n* = 13) or due to the large variation in costs between the 13 observations of each method as shown in the large standard deviations (Table 6). Similar studies should be undertaken to be able to calculate the cost effectiveness of such agents in terms of QALY so as to provide to the health payer, with comparative data of the cost effectiveness of this clinically effective treatment. Biologic registers are of uttermost importance for such evaluations as they can provide long-term data on TNF-*α* antagonists and facilitate the path to model building for the calculation of QALY [28]. This data, which is currently unavailable in Malta, could shed a more decisive light on the timely intervention of TNF-*α* antagonists which could ultimately result in a clinically effective, yet cost-effective treatment. 

## 5. Conclusion

The economic burden of the biological agents and the use of TNF-*α* antagonists in rheumatology is many times questioned by policy makers and financial settings within a national health service scheme. An improvement in quality of life is achieved within 6 months of therapy, as shown in this short-term study, and if this is sustained, the magnitude of the pharmacoeconomic impact increases substantially. This initial data could be useful to transmit the message to policy makers that for each individual patient, the potential of improving the quality of life and the pharmacoeconomic benefit is evident. Long-term benefit of biological treatment can hence be evaluated through further assessments.

## Figures and Tables

**Table 1:** Analysis of HAQ scores.

Daily activities (HAQ)	Phase	Difference in mean	*P* values*
Dressing and grooming	Phase 1-Phase 2	0.96	0.00
	Phase 1-Phase 3	1.00	0.00
	Phase 2-Phase 3	0.04	0.98

Rising	Phase 1-Phase 2	1.08	0.00
	Phase 1-Phase 3	1.23	0.00
	Phase 2-Phase 3	0.15	0.62

Eating	Phase 1-Phase 2	1.18	0.00
	Phase 1-Phase 3	1.26	0.00
	Phase 2-Phase 3	0.08	0.91

Walking	Phase 1-Phase 2	1.12	0.00
	Phase 1-Phase 3	1.27	0.00
	Phase 2-Phase 3	0.15	0.75

Hygiene	Phase 1-Phase 2	0.97	0.00
	Phase 1-Phase 3	1.03	0.00
	Phase 2-Phase 3	0.05	0.97

Reach	Phase 1-Phase 2	0.96	0.00
	Phase 1-Phase 3	1.12	0.00
	Phase 2-Phase 3	0.15	0.83

Grip	Phase 1-Phase 2	1.10	0.00
	Phase 1-Phase 3	1.23	0.00
	Phase 2-Phase 3	0.13	0.80

Activities	Phase 1-Phase 2	1.44	0.00
	Phase 1-Phase 3	1.46	0.00
	Phase 2-Phase 3	0.03	0.98

The difference in mean scores obtained between phases for every activity assessed within the HAQ and their relative level of significance (significance noted at *P* < 0.05*).

**Table 2:** Analysis of SF-36 scores.

SF-36 domain	Phase	Difference in mean (%)	*P* value*
Physical function (PF)	Phase 1-Phase 2	36.54	0.00
	Phase 1-Phase 3	38.46	0.00
	Phase 2-Phase 3	1.92	0.96

Physical role (PR)	Phase 1-Phase 2	69.23	0.00
	Phase 1-Phase 3	73.08	0.00
	Phase 2-Phase 3	3.85	0.94

Emotional role (ER)	Phase 1-Phase 2	64.07	0.00
	Phase 1-Phase 3	79.46	0.00
	Phase 2-Phase 3	15.40	0.20

Bodily pain (BP)	Phase 1-Phase 2	51.62	0.00
	Phase 1-Phase 3	58.62	0.00
	Phase 2-Phase 3	7.00	0.48

Vitality (V)	Phase 1-Phase 2	41.92	0.00
	Phase 1-Phase 3	44.23	0.00
	Phase 2-Phase 3	2.31	0.85

Social functioning (SF)	Phase 1-Phase 2	54.81	0.00
	Phase 1-Phase 3	56.73	0.00
	Phase 2-Phase 3	1.92	0.93

Mental health (MH)	Phase 1-Phase 2	32.62	0.00
	Phase 1-Phase 3	34.15	0.00
	Phase 2-Phase 3	1.54	0.97

General health (GH)	Phase 1-Phase 2	25.08	0.00
	Phase 1-Phase 3	26.85	0.00
	Phase 2-Phase 3	1.77	0.90

The difference in mean scores obtained between phases for every SF-36 domain and their relative level of significance (significance noted at *P* < 0.05*).

**Table 3:** Resulting ICERs in terms of improvement in the HAQ.

Patients	Cost differences between Treatment A and B	Difference in HAQ score between Treatment A and B	ICER per unit of improvement in HAQ over 6 months	ICER per 10% improvement in the HAQ over 6 months
Patient 1	€6,451	0.85	€7,589	€2,277
Patient 2	€5,218	0.65	€8,028	€2,408
Patient 3	€5,444	2.15	€2,532	€759.6
Patient 4	€6,489	1.00	€6,489	€1,947
Patient 5	€5,302	0.90	€5,891	€1,767
Patient 6	€6,536	1.05	€6,225	€1,868
Patient 7	€6,476	1.15	€5,631	€1,689
Patient 8	€4,891	2.30	€2,127	€638.1
Patient 9	€5,705	1.30	€4,388	€1,316
Patient 10	€6,508	0.25	€26,030	€7,809
Patient 11	€5,649	1.50	€3,766	€1,130
Patient 12	€6,325	1.10	€5,750	€1,725
Patient 13	€1,802	1.50	€1,201	€360
Average	**€5,600**	**1.20**	**€6,588**	**€1,977**

The ICERs per unit improvement and per 10% improvement in the HAQ following 6 months of TNF-*α* inhibitor therapy.

**Table 4:** Resulting ICERs in terms of improvement by the DAS-28.

Patients	Cost differences between Treatment A and B	Difference in DAS-28 score between Treatment A and B	ICER per unit of improvement in the DAS-28 over 6 months	ICER per 10% improvement in the DAS-28 over 6 months
Patient 1	€6,451	3.72	€1,734	€1,630
Patient 2	€5,218	3.88	€1,345	€1,264
Patient 3	€5,444	3	€1,815	€1,706
Patient 4	€6,489	3.46	€1,875	€1,763
Patient 5	€5,302	1.79	€2,962	€2,784
Patient 6	€6,536	2.51	€2,604	€2,448
Patient 7	€6,476	3.25	€1,993	€1,873
Patient 8	€4,891	2.88	€1,698	€1,596
Patient 9	€5,705	2.16	€2,641	€2,483
Patient 10	€6,508	1.49	€4,368	€4,106
Patient 11	€5,649	1.89	€2,989	€2,810
Patient 12	€6,325	3.27	€1,934	€1,818
Patient 13	€1,802	2.01	€897	€843
Average	**€5,600**	**2.71**	**€2,220**	**€2,087**

The ICERs per unit improvement and per 10% improvement in the DAS-28 following 6 months of TNF-*α* inhibitor therapy.

**Table 5:** Resulting ICERs in terms of improvement in the SF-6D.

Patient number	Cost differences between Treatment A and B	Difference in SF-6D between Treatment A and B	ICER per unit of improvement in the SF-6D	ICER per 10% improvement in the SF-6D
Patient 1	€6,451	0.35	€18,431	€1,843
Patient 2	€5,218	0.36	€14,494	€1,449
Patient 3	€5,444	0.22	€24,745	€2,475
Patient 4	€6,489	0.19	€34,152	€3,415
Patient 5	€5,302	0.18	€29,456	€2,946
Patient 6	€6,536	0.31	€21,084	€2,108
Patient 7	€6,476	0.18	€35,978	€3,598
Patient 8	€4,891	0.19	€25,742	€2,574
Patient 9	€5,705	0.19	€30,026	€3,003
Patient 10	€6,508	0.24	€27,117	€2,712
Patient 11	€5,649	0.37	€15,268	€1,527
Patient 12	€6,325	0.32	€19,766	€1,977
Patient 13	€1,802	0.37	€4,870	€487
Average	**€5,600**	**0.27**	**€23,164**	**€2,316**

The ICERs per unit improvement and per 10% improvement in the SF-6D following 6 months of TNF-*α* inhibitor therapy.

**Table 6:** Statistical analysis between the choice of health outcome used to express ICERs.

Health benefit outcome	Mean (€)	*P* value	SD
HAQ	1976.469		1860.4
DAS-28	2086.469	0.789	837.3
SF-6D	2316.376		869.3

The level of significance (significance noted at *P* < 0.05) and standard deviations between the HAQ, DAS-28, and the SF-6D.
